# Supplementary material for: Comparative multiomics analysis of cell physiological state after culture in a basket bioreactor
Source: Sci Rep. 2022 Nov 23;12:20161. doi: 10.1038/s41598-022-24687-4 (PMC9686226; doi:10.1038/s41598-022-24687-4)
Supplement: Supplementary file 1 — Supplementary Information 1. [file 41598_2022_24687_MOESM1_ESM.zip › raw data/Metabolomics raw data/5.MetKeggEnrichment/EG.vs.CG/EG.vs.CG_neg_KEGG_map/KEGG.map.html]

Pathway Enrichment


  

# The most enriched pathway terms of EG.vs.CG\_neg

Statistic method: hypergeometric test

FDR correction method: Benjamini and Hochberg

| MapID | MapTitle | Pvalue | AdjustedPv | x | y | n | N | EnrichDirect | GeneIDs |
| --- | --- | --- | --- | --- | --- | --- | --- | --- | --- |
| map01523 | Antifolate resistance | 0.0773405698778833 | 0.953867028493894 | 2 | 2 | 19 | 67 | Over | Com\_1314\_neg Com\_585\_neg |
| map00061 | Fatty acid biosynthesis | 0.191566642312911 | 0.953867028493894 | 2 | 3 | 19 | 67 | Over | Com\_8728\_neg Com\_9838\_neg |
| map00330 | Arginine and proline metabolism | 0.191566642312911 | 0.953867028493894 | 2 | 3 | 19 | 67 | Over | Com\_41\_neg Com\_9938\_neg |
| map00140 | Steroid hormone biosynthesis | 0.283582089552239 | 0.953867028493894 | 1 | 1 | 19 | 67 | Over | Com\_3385\_neg |
| map00524 | Neomycin, kanamycin and gentamicin biosynthesis | 0.283582089552239 | 0.953867028493894 | 1 | 1 | 19 | 67 | Over | Com\_9\_neg |
| map00592 | alpha-Linolenic acid metabolism | 0.283582089552239 | 0.953867028493894 | 1 | 1 | 19 | 67 | Over | Com\_5351\_neg |
| map00920 | Sulfur metabolism | 0.283582089552239 | 0.953867028493894 | 1 | 1 | 19 | 67 | Over | Com\_1828\_neg |
| map04212 | Longevity regulating pathway - worm | 0.283582089552239 | 0.953867028493894 | 1 | 1 | 19 | 67 | Over | Com\_8728\_neg |
| map04931 | Insulin resistance | 0.283582089552239 | 0.953867028493894 | 1 | 1 | 19 | 67 | Over | Com\_9\_neg |
| map05020 | Prion diseases | 0.283582089552239 | 0.953867028493894 | 1 | 1 | 19 | 67 | Over | Com\_3385\_neg |
| map01040 | Biosynthesis of unsaturated fatty acids | 0.395200602770632 | 0.953867028493894 | 3 | 7 | 19 | 67 | Over | Com\_1620\_neg Com\_5321\_neg Com\_8728\_neg |
| map00051 | Fructose and mannose metabolism | 0.489823609226594 | 0.953867028493894 | 1 | 2 | 19 | 67 | Over | Com\_20\_neg |
| map00120 | Primary bile acid biosynthesis | 0.489823609226594 | 0.953867028493894 | 1 | 2 | 19 | 67 | Over | Com\_1828\_neg |
| map00340 | Histidine metabolism | 0.489823609226594 | 0.953867028493894 | 1 | 2 | 19 | 67 | Over | Com\_3926\_neg |
| map00360 | Phenylalanine metabolism | 0.489823609226594 | 0.953867028493894 | 1 | 2 | 19 | 67 | Over | Com\_267\_neg |
| map04742 | Taste transduction | 0.489823609226594 | 0.953867028493894 | 1 | 2 | 19 | 67 | Over | Com\_267\_neg |
| map04923 | Regulation of lipolysis in adipocytes | 0.489823609226594 | 0.953867028493894 | 1 | 2 | 19 | 67 | Over | Com\_3385\_neg |
| map05230 | Central carbon metabolism in cancer | 0.489823609226594 | 0.953867028493894 | 1 | 2 | 19 | 67 | Over | Com\_585\_neg |
| map00190 | Oxidative phosphorylation | 1 | 1 | 1 | 3 | 19 | 67 | Over | Com\_345\_neg |
| map00240 | Pyrimidine metabolism | 1 | 1 | 1 | 3 | 19 | 67 | Over | Com\_1314\_neg |
| map00270 | Cysteine and methionine metabolism | 1 | 1 | 1 | 3 | 19 | 67 | Over | Com\_585\_neg |
| map00350 | Tyrosine metabolism | 1 | 1 | 1 | 3 | 19 | 67 | Over | Com\_7379\_neg |
| map00430 | Taurine and hypotaurine metabolism | 1 | 1 | 1 | 3 | 19 | 67 | Over | Com\_1828\_neg |
| map00520 | Amino sugar and nucleotide sugar metabolism | 1 | 1 | 1 | 3 | 19 | 67 | Over | Com\_9\_neg |
| map00770 | Pantothenate and CoA biosynthesis | 1 | 1 | 1 | 3 | 19 | 67 | Over | Com\_1208\_neg |
| map02010 | ABC transporters | 1 | 1 | 1 | 3 | 19 | 67 | Over | Com\_1828\_neg |
| map04728 | Dopaminergic synapse | 1 | 1 | 1 | 3 | 19 | 67 | Over | Com\_7379\_neg |
| map04925 | Aldosterone synthesis and secretion | 1 | 1 | 2 | 7 | 19 | 67 | Over | Com\_3385\_neg Com\_345\_neg |
| map04978 | Mineral absorption | 1 | 1 | 1 | 3 | 19 | 67 | Over | Com\_585\_neg |
